# Prospective User Perceptions and Acceptability of a Wearable Fetal Heart Monitor: Cross-Sectional Survey

**DOI:** 10.2196/85959

**Published:** 2026-08-10

**Authors:** Nikki Keene Woods, Anna Brake, Salsabila Attaria, Yongkuk Lee, Jolynn Dowling, Jamie Harrington

**Affiliations:** 1 College of Health Professions Wichita State University Wichita, KS United States; 2 College of Engineering Wichita State University Wichita, KS United States

**Keywords:** fetal heart monitor, maternal health, fetal health, telehealth, remote monitor, acceptability

## Abstract

**Background:**

Limited access to prenatal care, particularly in maternity care deserts and other underserved communities, contributes to maternal and fetal health disparities. Advances in telehealth and wearable fetal monitoring technologies, including fetal electrocardiography, offer opportunities to support remote fetal assessment and supplement traditional prenatal care. Understanding the acceptability of these technologies among potential users is important for informing future device development and implementation.

**Objective:**

This study aims to evaluate perceptions of acceptability and user preferences regarding a wireless fetal heart monitor among women of reproductive age.

**Methods:**

A survey was designed to assess the acceptability and user preferences of a wireless fetal monitoring device among women of reproductive age to guide device development. The survey was administered using the Qualtrics XM online survey platform. The survey was distributed using snowball recruitment through community and online postering. Compensation was offered to survey respondents. Data were analyzed using SPSS (version 29).

**Results:**

A total of 163 participants completed the survey, with 103 responses to acceptability questions. Older women aged 30 to 49 years showed higher rates of acceptability (40/54, 74.1%) than young women aged 18 to 29 years (26/49, 53.1%). In total, 70.7% (73/103) of the participants preferred a device measuring 2.5 × 7.6 cm or smaller.

**Conclusions:**

Designing a device that incorporates patient preferences may increase the likelihood of patient adoption and consistent use. This study examined perceived acceptability of a hypothetical monitoring device, providing a preliminary foundation to inform future device development and acceptability assessment.

## Introduction

### Background

In the United States, standard prenatal care recommendations include a health care provider visit every 4 weeks until 28 weeks’ gestation, every 2 weeks until 36 weeks’ gestation, and weekly from 36 weeks’ gestation until birth. Research shows that telehealth has been used to supplement prenatal care in areas with limited health care access, and it has been shown that patients who are able to have a combination of virtual and in-person health care provider visits over the course of their obstetrics care have higher satisfaction levels with their care [1,2]. Patients who require childcare or travel long distances to receive care also benefit from a telehealth model, as it allows them to receive health care on a more consistent basis [3]. A fetal heart monitoring device could be used as a supplementary method of fetal monitoring for those patients who are not able to access a traditional clinic easily or who may need to be monitored more frequently. This could be a more feasible option, bridging the access gap in areas with limited antenatal health care access.

Fetal monitoring is used throughout pregnancy to assess fetal well-being and oxygenation status. Doppler ultrasound technology, first introduced for clinical fetal monitoring in the 1970s, remains one of the most commonly used tools during both routine prenatal care and labor. The device operates by emitting ultrasound waves through a handheld transducer; differences between the transmitted and reflected wave frequencies are used for analysis (the Doppler shift) [4]. This allows clinicians to evaluate the movement of fetal cardiac walls and valves, providing real-time information about fetal heart activity. Over the past several decades, the use of Doppler ultrasound has contributed to earlier detection of fetal compromise and improved perinatal outcomes, particularly when incorporated into standardized antenatal care monitoring protocols [5,6].

Unlike intermittent Doppler ultrasound, which is typically used during clinic visits, fetal electrocardiogram (fECG) enables longer-duration monitoring by capturing electrical cardiac signals [7,8]. As Doppler technology is not used for continuous fetal monitoring due to risk of harming the fetus due to overheating, fetal monitoring tools such as fECG may be safer alternatives for continuous monitoring [9,10]. The fECG comprises electrical signals that can be sensed using noninvasive sensors that are placed on the abdomen of a pregnant patient. The electrical signals that comprise the fECG are very similar to those that comprise the adult echocardiogram heart rhythms, but they create a weaker signal. As the fECG signals are weaker, algorithms are used to separate the fECG signals from the maternal and bodily noise signals [11].

While fECG has demonstrated feasibility for fetal heart rate detection, its clinical effectiveness and role in routine prenatal care are still being evaluated. Remote fECG monitoring should therefore be viewed as a complementary approach rather than a replacement for established antenatal assessment methods.

### Health Disparities and Antepartum Care

A significant factor contributing to antepartum care health disparities is the prevalence of maternity care deserts, where there is limited or no access to obstetrical services or health care providers [12]. Low reimbursement rates, financial issues, and lack of obstetrical and anesthesia providers have forced many hospitals to close their obstetrical units. Those who are pregnant are sometimes forced to drive longer distances to access prenatal services, which also contributes to poor outcomes [13-15]. Additionally, individuals living in rural and underserved urban communities generally face worse health outcomes than those in well-resourced urban areas [16-18]. This disparity may be driven by limited access to health care, unemployment, financial hardship, and educational inequities [5].

Maternal health outcomes are worse in rural areas, and fetal outcomes are also affected. [19]. Unfortunately, when maternal health outcomes during pregnancy are worsened, this can adversely affect fetal health outcomes during pregnancy. Maternal health issues that are directly related to pregnancy (preeclampsia, gestational diabetes, etc) and underlying chronic health issues are some of the top contributors to maternal and fetal morbidity and mortality [20].

Fetal monitoring is a vital aspect of antepartum care. Technology in this area has greatly advanced in the previous decades [21]. Current fetal monitoring techniques, such as Doppler monitoring, can be bulky and restrict maternal movement during the monitoring period. During Doppler monitoring, the device is fastened to the maternal abdomen using an elastic band. It is important that the device be applied with care to optimize the accuracy of readings [22]. One thing to note about Doppler monitoring is that during the monitoring period, the patient is required to be immobile to increase the accuracy of readings [23].

### Importance of Early Detection of Congenital Heart Defects

During the third week of pregnancy, the fetal heart begins to develop. By the seventh week, it has developed into its structure of 4 chambers. The fetal heart rate can be detected by Doppler ultrasound beginning at 10 weeks of pregnancy. Routine monitoring of the fetal heart begins around 18 weeks of pregnancy [5].

Congenital heart defects (CHDs) are the foremost factor leading to fetal mortality from age 0 to 1 year [24]. They often impact the individual in a lifelong manner, and some are more complex than others, needing advanced treatment techniques and accurate monitoring. Ventricular septal defects, which cause heart malformation, are the most common type of defect, impacting 1% of babies born per year [24,25]. Ventricular septal defects cause a hole between the ventricles of the heart; they can exist by themselves, or they can comprise one of multiple CHDs affecting a patient [26]. CHD treatment has improved in recent years, and an important contributing factor to this improvement has been early detection. Early detection is essential, as it allows health care providers to create a treatment plan and monitor the baby before the baby is critically affected by the defect [5,27].

Difficulties in accessing care in rural areas can lead to lower rates of CHD detection in rural areas compared to urban areas [28]. Inability to access maternal fetal medicine specialists in rural areas can also impede early diagnosis. To address this health care gap and increase access to maternal and antenatal health care, remote monitoring may be a feasible alternative to allow health care providers to screen earlier for CHD and monitor higher-risk patients more frequently.

### Telehealth

Telehealth services include any health care experience that is aided by telecommunication (video or audio communication) [29]. Telehealth is currently used in a variety of ways to enhance obstetrics care through apps that allow for services such as health care diary entries, remote blood pressure monitoring, and fertility tracking. Numerous studies have also explored the practice of remote fetal monitoring during antepartum care [30,31]. Remote monitoring and telehealth virtual visits with health care providers can help to increase patient access to care and reduce the need for in-person health care appointments [2,32].

During the COVID-19 pandemic, telehealth use greatly increased; however, this increase was more profound in urban communities than in rural communities. Barriers such as internet access and less widespread use of telehealth in rural communities have led to people in rural areas not fully benefiting from this technology [29]. In the future, telehealth can be used to bridge the rural health care access gap, allowing for increased rates of maternal and perinatal health care [33].

Remote fetal monitoring technologies have been proposed as a potential adjunct to prenatal care, particularly in settings where access to in-person services is limited [34]. Rather than directly preventing maternal mortality, such technologies may support earlier detection of fetal concerns, facilitate monitoring in higher-risk pregnancies, and reduce logistical burdens associated with frequent clinic visits [30,35,36]. In several experimental trials where remote and in-hospital fetal monitoring technologies were compared, participants in the remote fetal monitoring groups voiced higher levels of satisfaction; additionally, health outcome differences between the hospital and remote groups were not statistically significant, indicating that remote fetal monitoring may provide a viable supplement to in-person clinical monitoring [37,38].

### Study Aim

This study aimed to evaluate the acceptability of a wireless fetal heart monitor among women of reproductive age for monitoring fetal heart rate throughout pregnancy. A wireless fetal heart monitor could allow mothers to be more comfortable and feel more in control during their prenatal monitoring sessions. This wireless fetal monitor would be placed on the maternal abdomen. The device would use an algorithm to extract the fetal heart data from the maternal heart data and bodily noise. The wireless fetal heart monitor would use fECG technology to monitor the fetal heart signals. This technology differs from techniques such as Doppler ultrasound that are traditionally used during fetal monitoring sessions. As previously discussed, it collects the electrical signals from the maternal abdomen. These signals are separated using a standardized algorithm, allowing the extracted fECG to be analyzed independently of the maternal ECG [11].

This extracted fECG would be transmitted via an app to health care providers, with the goal of allowing for increased health care accessibility and monitoring of the fetal heart. The wireless fetal monitor could allow women who have low health care access to have more consistent care. The device is intended to be multifunctional to match the needs of the patient population and clinical setting, as it could be used for both continuous monitoring and intermittent use, as well as in multiple settings, including both in-patient and home-based care. To inform device development, a survey was designed, distributed, and analyzed among women of reproductive age. Although participation was not limited to individuals who were currently pregnant, the broader population was included to provide a foundational understanding to support future research and device development. The questionnaire was developed to address the question “What is the level of acceptability of a fetal monitoring device among women of reproductive age based on survey data?”

The questionnaire complements concurrent research on the development of a wearable fetal monitoring device by helping to determine the optimal device size and specifications. This allows for the development of a device that best serves the target population and will be practical for everyday use.

## Methods

### Survey Development

#### Pilot Survey

The questionnaire was informed by prior research on acceptability and usability of wearable health technologies and refined through a pilot survey assessing readability, flow, and comprehension [39-41]. While formal psychometric validation was not conducted, pilot testing and literature grounding supported the face validity of acceptability measures for this formative study.

Before implementation, a pilot survey was created and distributed. The pilot survey allowed the survey to be tested for readability and flow. Participants were asked to complete the survey and provide feedback. Participant acceptability levels and opinions on the device were not evaluated; instead, at the end of the pilot survey, participants were asked to provide feedback on the survey (length, readability, etc). All feedback was anonymous. Improvements were made based on participant feedback. Metrics provided by the survey software Qualtrics XM (Qualtrics) were also examined.

Pilot survey participants indicated that the survey was of a reasonable length. Most completed the survey on a mobile phone; to best accommodate this mode of survey completion, the survey was assessed to ensure readability on mobile phones and laptops. Definitions and clarifications were added to the survey to provide improved readability, accessibility, and ease of participation. After the pilot survey feedback was analyzed and added to the survey, the updated survey was launched.

#### Survey

The questionnaire assessed perceived acceptability of a wearable fetal heart monitoring device among women of reproductive age, representing potential future users rather than exclusively pregnant individuals.

The Qualtrics survey software was used to build an online questionnaire to assess acceptability perceptions of the device. The questionnaire included 47 questions, implementing the 5-point Likert scale, multiple-choice questions, and 1 open-ended question at the end. Scaled items used the following response options: strongly disagree, somewhat disagree, neither agree nor disagree, somewhat agree, and strongly agree. Participants accessed the survey via a survey link through a QR code. All survey responses were anonymous.

### Survey Structure

#### Consent

Before participants began the survey, an informed consent form was provided at the beginning of the survey. Participants were also informed of the estimated duration of the survey. This time estimate was based on results from the pilot survey. Question types and topics (including age, gender, educational status, health status, internet access, and technology) were shared, and data security information was provided to participants.

#### Background Information

To ensure that participants were informed about the fetal monitoring device that the survey was assessing, an explanation of the device and a photo of a monitoring device were provided: “This survey is about wearable medical devices. These monitoring devices collect data on your heart rate, blood pressure, and if you were pregnant could collect your baby’s heart rate and movement. Below is a picture of the medical device. It is about the same size and weight as a band-aid and could be worn for one week. If used during pregnancy, it would be worn on your abdomen or belly and could send your health data to your doctor/health care provider.”

#### Conditional Logic

To ensure that participants fit the survey requirements, conditional logic was used for several questions. Conditional logic allowed for survey responses that did not fit survey parameters (female, aged 18-49 years) to be discarded. When survey participants gave a response that was outside survey parameters, the survey was automatically ended, and participants did not complete the following survey questions.

Several screening questions were used to determine whether participants met the eligibility criteria for the survey: participants were required to consent to the survey and use of their anonymized information, and they were required to be a female aged 18 to 49 years. Conditional logic ensured that qualifying participants were included in the survey data.

#### IT Information

The definition of the term “information technology” was provided to increase the readability and understandability of the survey: “Information technology is the use of computers to collect, store and share data.”

Participants were assessed on their IT experience using a 5-point Likert scale. Statements were provided, and participants indicated their level of agreement with the statement. For example, statements such as “I think that information technology has some benefits” or “I think that privacy breaches are a serious issue today” were provided, and participants indicated their level of agreement or disagreement with the provided statements.

#### Acceptability

Participant acceptability of the wearable device was measured with 5 questions assessed on a 5-point Likert scale ranging from “extremely unlikely” to “extremely likely.” These questions were used to gauge perceived device acceptability levels across common use contexts, including comfort with wearing the device daily and likelihood of using the device at home to monitor fetal movement and fetal heart rate, during prenatal care appointments, and during labor. For analysis, responses of “extremely likely” and “likely” were coded as acceptable, while all other responses were coded as not acceptable. Each item was analyzed as a dichotomous outcome, and associations between participant characteristics and acceptability were evaluated using the chi-square test.

#### Health Literacy

Health literacy was assessed using 3 questions adapted from the US Centers for Disease Control and Prevention guidelines. These items measured participants’ perceived difficulty in finding medical information, understanding information communicated by health professionals, and understanding written health information [42,43].

Responses were recorded on a 6-point scale ranging from extremely easy (score=4), somewhat easy (score=3), somewhat difficult (score=2), very difficult (score=1), I do not look for health information (score=0), and I do not pay attention to written health information (score=0). As 3 questions were used to determine health literacy, with a maximum additive score of 12 and a minimum score of 0, health literacy levels were tabulated using the cumulative score [44]. Participants were separated into 2 categories: those who have high health literacy and those who have less than high health literacy, consistent with prior approaches to categorizing self-reported health literacy and the Centers for Disease Control and Prevention guidelines [43]. Associations between health literacy (high vs less than high) and device acceptability (acceptable vs not acceptable) were examined using the chi-square test.

### Recruitment

Responses were collected via snowball recruitment of participants. The study aim, along with a QR code linking to the questionnaire, was disseminated through posters in public spaces and electronic newsletters. Participant inclusion criteria included being a woman of reproductive age (18-49 years) and the ability to read English. Responses were collected from September 2023 to January 2024.

An incentive of US $20 in the form of a prepaid gift card was provided to participants who completed the survey. To maintain anonymity of survey respondents, compensation information was not collected with participant responses. At the end of the survey, a link was provided to all respondents. Instructions were provided to participants indicating that participants who desired to receive compensation for their survey completion could follow the link to a compensation survey. This survey was separate from the acceptability survey and allowed for participant responses to maintain anonymity. Participants who filled out the compensation survey were provided with compensation, which was a prepaid gift card that was mailed to participants. Follow-up was conducted to ensure that participants received compensation.

### Statistical Analysis

Survey responses were analyzed using SPSS Statistics (version29; IBM Corp). Descriptive statistics were used to summarize participant characteristics and survey responses. Associations between health literacy and device acceptability, as well as between age group and acceptability, were examined using the chi-square test. Qualitative responses to the open-ended survey question were analyzed using thematic analysis to identify key themes.

Participant location (urban or rural) was determined using the submission IP address. The zip code location of the IP address was determined using an IP geolocation tool [45]. The urban or rural designation of the zip code was found using the Rural Health Information Hub. Counties with a population of 5000 or fewer inhabitants were considered rural for the purposes of the survey analysis [46]. Responses were individually analyzed to determine the classification of their locations.

### Data Exclusion

Of the initial responses received, incomplete surveys and responses identified as automated or invalid were excluded prior to analysis. Only surveys meeting eligibility criteria and completing acceptability items were included in the final analyses.

### Ethical Considerations

This study was reviewed and approved by the Wichita State University Institutional Review Board (institutional review board number: 5260). Informed consent was obtained electronically prior to survey participation. Survey responses were anonymous, and no identifying information was collected. Data were stored securely and analyzed in deidentified form. Participants received a US $20 prepaid gift card as compensation through a separate survey mechanism to preserve anonymity.

## Results

### Overview

A total of 163 participants completed the questionnaire. Demographic analyses were conducted using the full sample (N=163). Of these participants, 103 completed the device acceptability items and were included in acceptability analyses. On average, the survey participants completed it in 5.1 (SD 3.03) minutes, and 103 participants provided responses to questions gauging acceptability of the device. The total response numbers for each question varied slightly, as the questions were optional to complete.

### Demographics

The survey was limited to female participants aged 18 to 49 years. Participants’ age, ethnicity, and education level were collected. Participants’ internet access levels were also assessed. The median age of survey respondents was greater than 30 years (Table 1).

**Table 1:** Participant demographics (N=163).

		Participants, n (%)
Age group (years)		
	18-29	49 (30.1)
	30-49	114 (69.9)
Race or ethnicity		
	American Indian or Alaska Native	19 (11.4)
	Asian	12 (7.4)
	Black or African American	22 (13.4)
	Hispanic or Latinx	24 (14.8)
	Middle Eastern or North African	2 (1.3)
	White	81 (49.7)
	Two or more	3 (2)
Education		
	High school or equivalent	3 (2)
	Some college, no degree	69 (42.6)
	Associate degree	18 (10.8)
	Bachelor’s degree	35 (21.6)

The race or ethnicity breakdown for the survey participants included American Indian or Alaska Native: 11.4% (19/163), Asian: 7.4% (12/163), Black or African American: 13.4% (22/163), Hispanic or Latinx: 14.8% (24/163), Middle Eastern or North African: 1.3% (2/163), White: 49.7% (81/163), and 2 or more races or ethnicities: 2% (3/163). These percentages were similar to the percentage breakdown of the US population. The 2020 US Census recorded the following percentages for race and ethnicity: White: 57.8%, Black or African American: 12.1%, Hispanic or Latinx: 18.7%, American Indian or Alaska Native: 0.7%, Asian: 5.9%, 2 or more: 4.1%, and other: 0.6% [47]. The distributed survey included Middle Eastern or North African as a racial option. This designation was included with White in the 2020 Census, but it will be given its own designation in the 2030 Census [48].

The participant education levels were as follows: high school degree or equivalent: 2% (3/163), some college but no degree: 42.6% (69/163), associate degree: 10.8% (18/163), bachelor’s degree: 21.6% (35/163), and graduate degree: 23% (38/163). This is slightly higher than the US national average that was recorded in the 2020 Census [47].

### Health Literacy

Most participants demonstrated high health literacy (83/103, 80.6%), while 19.4% (20/103) were classified as having less than high health literacy. There was no statistically significant association between health literacy and device acceptability (*χ*^2^_1_=0.8; *P*=.34). Although a higher proportion of participants with high health literacy reported acceptability (55/83, 66.3%) compared to those with low health literacy (11/20, 55%), this difference was not statistically significant (*χ*^2^_1_=0.8; *P*=.34).

### Overall Device Acceptability

Among participants included in the acceptability analysis (n=103), participants aged 30 to 49 years demonstrated a higher rate of device acceptability (40/54, 74.1%) than younger participants aged 18 to 29 years (26/49, 53.1%; Table 2). A chi-square test of independence indicated a statistically significant association between age group and device acceptability (*χ*^2^_1_=4.9; *P*=.03), with participants aged 30 to 49 years demonstrating higher acceptability than those aged 18 to 29 years. Regarding device features, 70.7% (73/103) of participants preferred a device measuring 2.5 × 7.6 cm or smaller, indicating a preference for a smaller device. In total, 73.6% (120/163) of participants indicated that they would be comfortable with wearing the device daily. In gauging device convenience, 14.7% (24/163) strongly disagreed and 28.8% (47/163) somewhat disagreed that the monitor would limit their daily activities. Additionally, 49.7% (81/163) agreed to some degree that the device would be “compatible with most aspects of my activity.”

**Table 2:** Device acceptability by health literacy and age (n=103).

Variable			Acceptable, n (%)		Not acceptable, n (%)		*P* value	
Health literacy								.34
	Low	11 (55)		9 (45)				
	High	55 (66.3)		28 (33.7)				
Age group (years)								*.03* ^ a ^
	18-29	26 (53.1)		23 (46.9)				
	30-49	40 (74.1)		14 (25.9)				

^a^Italicized *P* value indicates statistical significance (*P*<.05).

When participants were asked about how they would feel about using the device for the first time, 11% (18/163) somewhat agreed and 2.5% (4/163) strongly agreed that they would feel anxious. However, 21.5% (35/163) strongly disagreed with feeling anxious about using a device they had not previously used. Over half of the respondents agreed that learning to use the device seemed easy.

### Participant Feedback

Responses to an open-ended question asking participants to voice doubts about the device indicated concern for privacy. Of the 23 participants who provided a written response, 6 participants voiced concern for data from the monitor being shared with third parties, insurance companies, or any entity outside of their care team. Participants stated concern about the process of learning more about how to use the device, as well as worry about potential side effects. Finally, concerns were raised about the accuracy of the device.

## Discussion

### Principal Findings

This formative survey examined perceived acceptability of a wearable fetal heart monitoring device among women of reproductive age. Overall, acceptability was moderate, with higher acceptance among older respondents and strong preferences for smaller, unobtrusive device designs.

As found in the survey, increasing acceptability levels were positively correlated with increasing age. A total of 163 participants completed the survey, with 103 responses to acceptability questions; older women aged 30 to 49 years showed higher rates of acceptability (n=40, 74.1%) than young women aged 18 to 29 years (n=26, 53.1%). Participant feedback involving concerns of the target population regarding the wearability, usability, and safety of the device will be considered when developing the fetal monitoring device.

### Interpretations

Overall acceptability was moderate to high, suggesting general openness to remote fetal monitoring technologies among the surveyed population. Higher acceptability observed among women aged 30 to 49 years compared to those aged 18 to 29 years may reflect greater perceived clinical value of monitoring or varying levels of trust in cloud-based storage and digital health technologies. Strong preference for a small and low-profile device highlights the importance of minimizing physical burden and visibility to support comfort and use.

Findings should be interpreted as reflecting perceptions of acceptability, not actual use or clinical effectiveness, as participants evaluated a hypothetical device rather than the actual technology. Furthermore, findings should be interpreted through a broad viewpoint, as the study population included all women of reproductive age, not specifically those who were pregnant at the time of the study or had been pregnant before. The perceptions of acceptability that were evaluated provide a foundation for more targeted acceptability research.

### Implications

When interpreting the questionnaire results, it should be noted that implications for device use and care in underserved areas are future oriented and were not empirically demonstrated in this study. Additionally, as the surveyed sample was not limited to those who were pregnant at the time of the survey or those who have had pregnancy experience, the results have decreased direct relevance to the target device users.

Assessing acceptability during early‑stage development provides critical guidance for user-centered design prior to feasibility, effectiveness, and implementation testing. Incorporating user-identified preferences, particularly related to device size and wearability, may increase adoption and sustained use of wearable fetal monitoring technologies. Age-related differences in acceptability suggest the need for tailored communication or education strategies to address perceived value and technology concerns among younger users.

Although not directly evaluated in this study, devices developed with user input may hold future potential to support prenatal care access in underserved or rural populations, supporting targeted investigation.

### Limitations

Most respondents were from urban areas (with a population >5000 residents) [46]. Given prior evidence of rural-urban differences in health care access and health literacy, the observed health literacy and device acceptability estimates may be higher than would have been observed in a sample with greater rural representation. People who live in rural locations have slower medical responses to health care emergencies such as stroke [16]. There are fewer specialists who work in rural areas, leading to a lack of proper health care availability [17]. In a study on rural health care use in the United States, patients residing in rural areas were generally found to have lower levels of health care use and worse health statuses than their urban counterparts [18]. All of these factors can contribute to rural health literacy levels, and this can create a reduced average health literacy level among patients residing in rural areas when compared to patients residing in urban areas [49].

Although remote fetal monitoring may be especially relevant in rural or underserved contexts, most respondents in this study resided in urban areas. As such, findings should be interpreted as reflecting general perceptions of acceptability rather than preferences specific to rural populations. The potential relevance of this technology for rural care represents a future application that warrants targeted investigation.

The survey included a highly educated population, and most respondents were from urban areas. This may have impacted the responses that were received, creating a skewed representation of anticipated device acceptability.

Survey distribution presented some challenges, as sharing the link online created a risk of bot-generated invalid responses. There was a survey that was implemented before this survey, but it was overwhelmed by bot responses, and the results were discarded in favor of creating a new survey and reimplementing it. Furthermore, the distribution methods used (snowball recruitment, postering, and social media) may not have created a fully representative sample of the target population.

As respondents were not required to be currently pregnant or to have recent pregnancy experience, findings reflect anticipated acceptability rather than experiential usability during pregnancy.

### Broader Implications and Future Directions

This questionnaire was one component of an interdisciplinary effort to develop a remote fetal monitoring device. The next phase of this project will involve prototype development and testing. Findings from the questionnaire are being used to inform device design, with the goal of ensuring it aligns with patient preferences and needs.

Future research would benefit from limiting respondents to pregnant individuals to more directly assess acceptability within the intended user population. The current survey also provides a foundation for refining future instruments that will evaluate user experience and acceptability during prototype testing.

As remote monitoring devices are introduced in clinical and home settings, patient education will be essential to successful adoption. Survey findings suggest that individuals with higher health literacy may be more receptive to using a fetal monitoring device, underscoring the importance of clear communication and patient-centered education strategies.

### Conclusions

These findings provide preliminary insight into perceived acceptability and patient-centered preferences for a hypothetical wearable fetal heart rate monitoring device among women of reproductive age. The results may inform early-stage design and device development by highlighting features related to usability, comfort, and convenience that are important to potential users. Participant feedback offers initial guidance for aligning device development with user expectations. However, these findings should be interpreted in light of the study’s limitations and as exploratory.

Future research is needed to evaluate acceptability among pregnant populations in real-world settings, as well as to examine how acceptability relates to actual device use and clinical outcomes.

## Abbreviations

CHD: congenital heart defect
fECG: fetal electrocardiogram
